# Effects of atomic-level nano-structured hydroxyapatite on adsorption of bone morphogenetic protein-7 and its derived peptide by computer simulation

**DOI:** 10.1038/s41598-017-15219-6

**Published:** 2017-11-09

**Authors:** Qun Wang, Menghao Wang, Xiong Lu, Kefeng Wang, Liming Fang, Fuzeng Ren, Guoming Lu

**Affiliations:** 1 Key Lab of Advanced Technologies of Materials, Ministry of Education, School of Materials Science and Engineering, Southwest Jiaotong University, Chengdu, 610031 Sichuan China; 2 College of Life Science and Biotechnology, MianYang Teachers’ College, Mianyang, 621006 Sichuan China; 3 National Engineering Research Center for Biomaterials, Genome Research Center for Biomaterials, Sichuan University, Chengdu, 610065 Sichuan China; 4 Department of Polymer Science and Engineering, School of Materials Science and Engineering, South China University of Technology, Guangzhou, 510641 China; 5 Department of Materials Science and Engineering, South University of Science and Technology of China, Shenzhen, Guangdong, 518055 China; 6 School of Computer Science and Engineering, University of Electronic Science and Technology of China, Chengdu, 610054 Sichuan China

## Abstract

Hydroxyapatite (HA) is the principal inorganic component of bones and teeth and has been widely used as a bone repair material because of its good biocompatibility and bioactivity. Understanding the interactions between proteins and HA is crucial for designing biomaterials for bone regeneration. In this study, we evaluated the effects of atomic-level nano-structured HA (110) surfaces on the adsorption of bone morphogenetic protein-7 (BMP-7) and its derived peptide (KQLNALSVLYFDD) using molecular dynamics and density functional theory methods. The results indicated that the atomic-level morphology of HA significantly affected the interaction strength between proteins and HA substrates. The interactions of BMP-7 and its derived peptide with nano-concave and nano-pillar HA surfaces were stronger than those with flat or nano-groove HA surfaces. The results also revealed that if the groove size of nano-structured HA surfaces matched that of residues in the protein or peptide, these residues were likely to spread into the grooves of the nano-groove, nano-concave, and nano-pillar HA, further strengthening the interactions. These results are helpful in better understanding the adsorption behaviors of proteins onto nano-structured HA surfaces, and provide theoretical guidance for designing novel bioceramic materials for bone regeneration and tissue engineering.

## Introduction

Hydroxyapatite [HA, Ca_10_(PO_4_)_6_(OH)_2_] is the major inorganic component of bones and teeth. As a bone implant material, HA has received increasing attention because of its exceptional interface or surface properties and excellent biocompatibility^1^. It is well-known that, *in vivo*, the HA surface is rapidly covered by proteins and other biomolecules, leading to the regulation of precipitated calcium phosphate crystal growth or alterations to crystal morphology^2^. Therefore, it is essential to study the surface properties of HA and interaction behaviors between HA and various proteins. Recently, numerous experimental and theoretical studies have been conducted to study the interaction mechanisms between HA and proteins, such as the bone morphogenetic protein-7 (BMP-7)^3^, BMP-2^4^, amelogenin^5^, osteopontin^6^, fibronectin^7^, bovine serum albumin (BSA)^8^, and sialoprotein^9^, among others. These studies suggest that the surface topology and composition, the pH values of protein solutions and mechanical properties of the biomaterials affect the interactions to some extent^10,11^. To achieve excellent biocompatibility and osteoinductivity, micro-nano structured HA surfaces were established.

Micro-structured HA generally resulted in cell morphology change and consequently altered cell function changes^12–15^. Henry *et al*.^16^ demonstrated that mouse pre-osteoblast cells (MC3T3-E1) were better arranged along micro-grooves with a groove width of 5 μm for the poly(ε-caprolactone) triacrylate/ HA substrates. Wang *et al*.^17^ also showed that the groove width of micro-patterned HA has a prominent effect on the orientated growth of osteoblast cells. Lu *et al*.^18^ found that HA micro-grooves with a width of 8 μm significantly affected contact guidance of osteoblasts and myoblasts, whereas those of 24 μm affected myoblasts only. Iwamoto *et al*.^19^ obtained a micrometer scale flat HA and nanometer scale convexo-concave HA and found that MC3T3-E1 and mouse fibroblast cells (L929) adhered to the flat HA surface but not to the convexo-concave HA surface.

Nanoscale HA also plays a significant role in regulating cell functions and adsorbing proteins. Wu *et al*.^20^ reported that the nano-HA porous structure is appropriate for blood vessels and fibrous connective tissue to enter, providing mesenchymal cells for implementing osteoinductivity of rhBMP-2 in the material. Jouve *et al*.^21^ indicated that the porous structure of nano-HA and “nanometer effect” of the nanomaterial significantly enhance HA surface area, provide good support for revascularization of the material, and formation of the bone matrix. Kasaj *et al*.^22^ demonstrated that nano-HA enhanced adhesion and proliferation in human periodontal ligament cells. Webster^23^ found that vitronectin adsorption has more obvious enhancement on nano-HA materials than that on micro-HA materials. Zhu *et al*.^24^ reported that the nano-HA/rhBMP-2 composite is more likely to stimulate new bone formation and had more excellent repairing ability for bone defect compared to the nano-HA.

BMP-7 is a member of the transforming growth factor-beta superfamily and comprises 112 residues. BMP-7 plays a crucial role in the induction and regulation of cartilage and bone formation and has an important influence on skeletal development and growth^25^. This protein has been widely examined in previous studies. Luo *et al*.^26^ reported that a lack of BMP-7 can result in murine skeletal defects. Schwarting *et al*.^27^ investigated the effects of BMP-7 on tendon bone repair and indicated that BMP-7 is extremely important for tendinous fibroblast transformation and differentiation at the tendon-bone interface. Cheng *et al*.^28^ suggested that BMP-7 promotes osteogenesis in mature osteoblasts. Tasnim *et al*.^29^ found that BMP-7 or BMP-2 improves the functional performance of primary human renal proximal tubule cells, particularly in the case of BMP-7. Zhou *et al*.^3^ used molecular dynamics (MD) and steered molecular dynamics (SMD) methods to investigate the adsorption mechanism of BMP-7 on HA (001) surfaces, and found that –COO– and -NH_2_ play vital roles in the adsorption of BMP-7 onto the HA surfaces by forming Ca-O connections and hydrogen bonds (H-bonds). Dong *et al*.^30^ investigated the dynamic behaviors of BMP-7 on HA (001) and HA (010) surfaces and found that both the type and number of differences of the adsorbed residues and interaction strengths are relevant to the specific texture of the HA surfaces.

BMP-7 not only has strong osteoinductivity, but also affects the growth and physiological function of the reproductive system. However, the structure of BMP-7 is complicated, it degrades easily in the body, and requires considerable time to be produced on a large scale. Thus, BMP-7-derived peptides have attracted attention because of their low-cost and excellent bio-functions^31^. Studies have shown that these peptides from the BMP-7 or BMP-7 receptors can be used to treat bone-related diseases. Kim *et al*.^32^ found that a novel bone forming peptide-1 (GQGFSYPYKAVFSTQ) from the BMP-7 has greater activity in osteogenic differentiation than that of whole BMP-7. Furthermore, the authors suggested that the BMP-7 peptide can also serve as a valuable adjuvant in medical therapies for bone-related diseases. Tao *et al*.^33^ prepared a new type of functional, self-assembling peptide nanofiber-hydrogel scaffold with the sequence RADKPS (ARG-ALA-ASP-LYS-PRO-SER); they clearly showed that RADKPS promoted cell proliferation and activity of human degenerative nucleus pulposus cells. Another peptide, KQLNALSVLYFDD (LYS-GLN-LEU-ASN-ALA-LEU-SER-VAL-LEU-TYR-PHE-ASP-ASP), is synthesized from the BMP-7 receptor I- and receptor II-binding active domains^31^ and, in addition, has excellent osteogenic activity and induces mineralization of bone marrow stromal stem cells. The structures of BMP-7-derived peptides are simple and easy to integrate into biomaterials while maintaining their biological activities during synthesis. Furthermore, these peptide molecules are small and the active sites can be easily exposed.

Although numerous studies have been devoted to the interactions between proteins and biological nanomaterials HA, it is nevertheless difficult to quantitatively comprehend the detailed interaction mechanisms involving nanomaterials and proteins by conventional experimental methodologies. Computer simulation is a favorable method to investigate such interaction mechanisms at the atomic and molecular levels. Therefore, here, we have utilized MD and density functional theory (DFT) simulation methods to investigate interactions between nanometer-scale HA and proteins/peptides, and focused on the effect of nano-morphology on protein/peptide adsorption. The HA (110) crystallographic surface was used to study the interaction with BMP-7 because it not only has high adsorption strength for molecules^34^, but also is one of the dominant surfaces observed in numerous experimental and theoretical studies^35–37^. The adsorption behaviors of BMP-7-derived peptide (KQLNALSVLYFDD) on these nano-structured HA (110) surfaces were also evaluated because of its excellent osteogenic activity. Our results are expected to advance understanding of the bioactivity mechanism of calcium phosphate biomaterials and offer theoretical guidance for HA applications in materials design and tissue engineering.

## Simulation Methods

### Building Models

**Figure 1:**
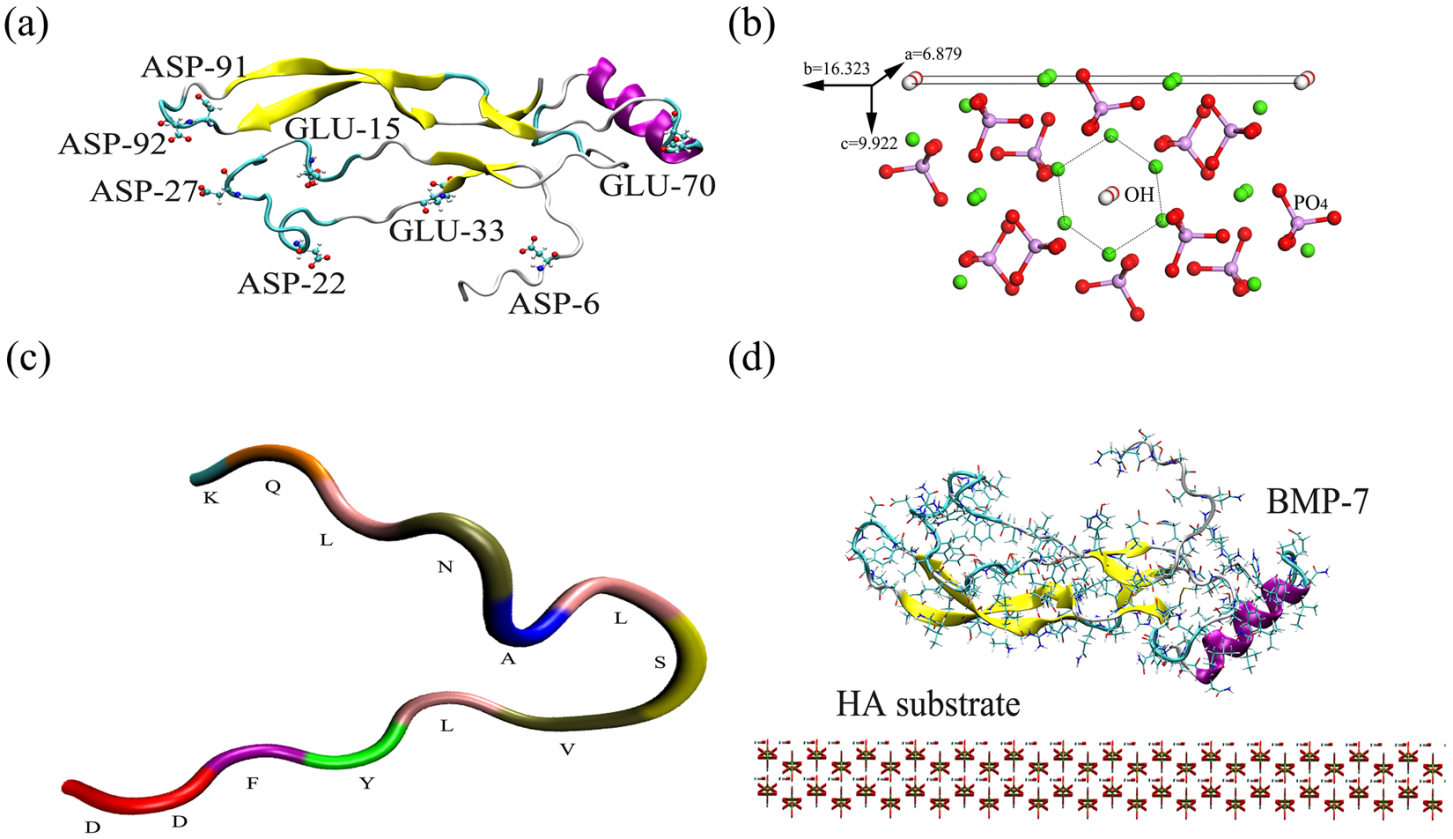
Newcartoon structures of (**a**) BMP-7 and (**c**) BMP-7-derived peptide (KQLNALSVLYFDD), (**b**) three-dimensional structure of HA (110) cell and (**d**) BMP-7 interacting with the flat HA surface before MD simulation.

The primary conformation of BMP-7 was obtained from the RCSB protein data bank (http://www.rcsb.org/pdb/, ID: 1M4U); the Newcartoon structure of BMP-7 is illustrated in Fig. 1a. BMP-7-derived peptide (KQLNALSVLYFDD) containing 13 amino acids was built according to the literature (Fig. 1c)^31^. BMP-7 and its derived peptide molecules were immersed in TIP3P water boxes and charges balanced with sodium and chloride ions. In order to obtain optimized geometries of BMP-7 and its derived peptide, a 20 ps energy minimization and a 1500 ps MD relaxation in the water box were performed with a time step of 0.5 fs in the NPT ensemble using NAMD package^38^. A constant temperature of 310.6 K and constant pressure of 101.3 kPa were used in the simulations, employing the modified Nose-Hoover method, in which Langevin dynamics was used to control fluctuations in the barostat.

**Figure 2:**
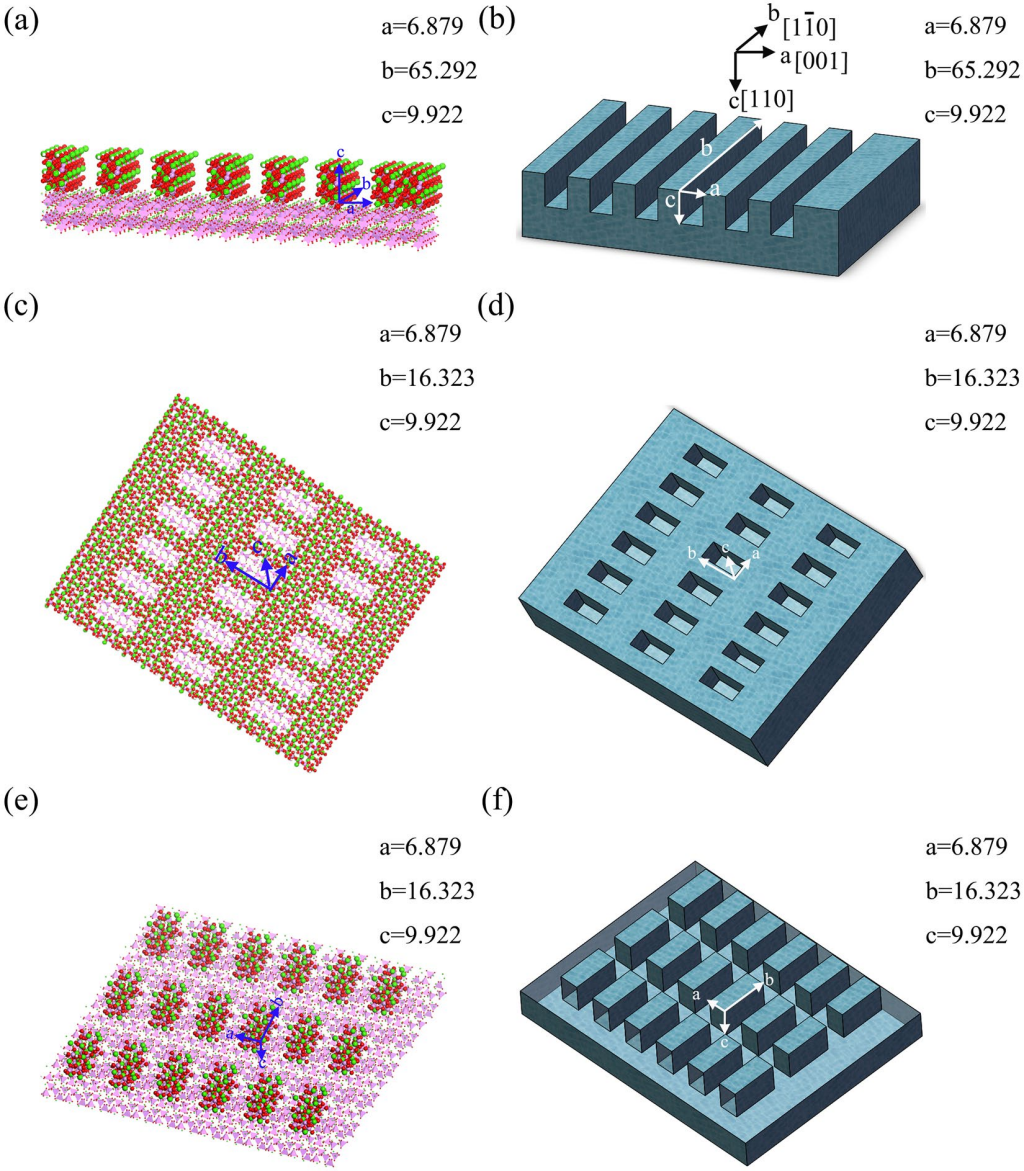
Structures of (**a**) and (**b**) groove1a-HA surfaces, (**c**) and (**d**) concave1a1b-HA surfaces, (**e**) and (**f**) pillar1a1b-HA surfaces. Note that the units of a, b, and c are Å.

The initial model of HA (*P*63/*m*) was acquired from the American Mineralogist Crystal Structure database^39^. The unit cell parameters of the HA crystal are *a = b = *9.424 Å and *c = *6.879 Å. The processes used to build nano-structured HA (110) surfaces were as follows. First, the flat HA (110) cell was built with dimensions of *a = *6.879 Å, *b = *16.323 Å, and *c = *9.922 Å (Fig. 1b), and then a flat HA (110) surface was constructed with a size of 96.288 × 65.292× 19.844 Å^3^. Finally, nine types of nano-structured HA (110) surfaces (three nano-grooves, three nano-concaves, and three nano-pillars) were prepared based on the flat HA (110) surface (Fig. 2). The nano-pillar HA (110) surfaces were similar to the nano-concave HA (110) surfaces except that the concaves became the pillars in the HA surfaces. The sizes and numbers of nano-grooves, nano-concaves, and nano-pillars of HA (110) are illustrated in Table 1. Note that only the HA (110) surface was present in this study, and hence the HA (110) surface was simplified as the HA surface. HA (110) surfaces with different terminations were also constructed, as shown in Supplementary Information. BMP-7 was laid on the flat HA surface (Fig. 1d), which illustrated the interaction between BMP-7 and the flat HA surface before MD simulation. The initial orientation of BMP-7 was chosen according to results from the literature^3^. The sizes of the interaction systems are shown in Table 1.

**Table 1:** Sizes of atomic-level nano-structured HA (110) and its interaction with BMP-7.

Models	Number of groove/concave/pillar	Volume of groove/concave/pillar (Å^3^)	increment of surface areas (Å^2^)
Groove1a-HA	6	6.879 × 65.292 × 9.922	6954.88
Groove2a-HA	4	13.758 × 65.292 × 9.922	4090.55
Groove3a-HA	3	20.637 × 65.292 × 9.922	2658.40
Concave1a1b-HA	18	6.879 × 16.323 × 9.922	8287.57
Concave2a1b-HA	12	13.758 × 16.323 × 9.922	7163.13
Concave2a2b-HA	8	13.758 × 32.646 × 9.922	7363.08
Pillar1a1b-HA	18	6.879 × 16.323 × 9.922	8287.57
Pillar2a1b-HA	12	13.758 × 16.323 × 9.922	7163.13
Pillar2a2b-HA	8	13.758 × 32.646 × 9.922	7363.08
Flat-HA-BMP-7		96.288 × 70.706 × 71.674	
Groove1a-HA-BMP-7		96.951 × 98.742 × 80.817	
Groove2a-HA-BMP-7		96.951 × 98.742 × 80.817	
Groove3a-HA-BMP-7		96.951 × 98.742 × 80.817	
Concave1a1b-HA-BMP-7		96.306 × 98.742 × 80.914	
Concave2a1b-HA-BMP-7		96.306 × 98.742 × 80.659	
Concave2a2b-HA-BMP-7		96.306 × 98.742 × 80.914	
Pillar1a1b-HA-BMP-7		96.306 × 98.742 × 80.914	
Pillar2a1b-HA-BMP-7		96.306 × 98.742 × 80.914	
Pillar2a2b-HA-BMP-7		96.306 × 98.742 × 80.914	

## Methods

All simulations were conducted with NAMD using CHARMM27 force field^40^ with the HA parameters added. Furthermore, three different nano-topographies of HA in our models used the same force field with the flat HA, because these nano-structured HA removed the geometric multiple of HA double cell atoms (Fig. 1b) and did not result in HA atoms/ions with different atomistic environments. Periodic boundary conditions were applied to all models. Particle-mesh Ewald summation was utilized to calculate the long-range electrostatic interactions with a cutoff distance of 14 Å to separate the direct and reciprocal space. Van der Waals interactions were cut off at 12 Å. The force field parameters for HA were fitted based on the results of Bhowmik, Hauptmann and Zhou^3,41,42^. Additionally, in order to maintain the stability of the systems, the combinational arrangements of protein and HA were firstly underwent 3000 ps MD simulations with a time step of 1 fs in the NPT ensemble. Then, 27000 ps MD simulations were conducted with a time step of 2 fs in the NVT ensemble. For simulation of the adsorption behavior of protein or peptide, tens of thousands of picoseconds simulations are considered suitable^43–45^.

The molecular graphics package VMD was employed to analyze the static and dynamic structure information after MD simulation. Snapshots of interaction systems, interaction energies, and interaction sites, among others, were acquired from VMD^46^.

The interaction energies were calculated to evaluate the interaction strength between proteins and the flat, nano-groove, nano-concave, and nano-pillar HA surfaces. The interaction energy (${E}_{{\rm{int}}}$) was calculated according to the equation (1)

1 $${E}_{{\rm{int}}}={E}_{HA+{\rm{BMP}}-7}\,-\,({E}_{HA}+{E}_{{\rm{BMP}}-7})$$

where ${E}_{{\rm{HA}}+{\rm{BMP}}-7}$,${E}_{{\rm{HA}}}$, and ${E}_{{\rm{BMP}}-7}$ represent the total interaction energy of the HA and protein, the energy of HA, and the energy of protein, respectively. Note that the more negative interaction energy indicates a greater interaction strength between the protein and HA^47^. The average interaction energies of the simulation systems over the final 100 ps are displayed in Table 2.

**Table 2:** Electrostatic and interaction energies between BMP-7 and HA surfaces.

Systems	ELEC Kcal·mol^−1^	Interaction energy Kcal·mol^−1^	Main absorbed residues after the MD simulation
**Flat-HA-BMP-7**	**−1883.96**	**−1873.59**	**ASP27 GLU70**
Groove1a-HA-BMP-7	−2053.36	−2029.57	ASP27 ASP91 GLU70
**Groove2a-HA-BMP-7**	**−2210.45**	**−2166.63**	**ASP27 ASP91 GLU70**
Groove3a-HA-BMP-7	−1580.31	−1560.77	ASP91 GLU33 VAL60
Concave1a1b-HA-BMP-7	−3171.04	−3177.8	ASP27 ASP92 LIE30 GLU70
**Concave2a1b-HA-BMP-7**	**−4720.71**	**−4660.49**	**ASP6 ASP27 ASP92 GLU1 GLU33**
Concave2a2b-HA-BMP-7	−3770.16	−3738.27	ASP6 ASP27 GLU33
Pillar1a1b-HA-BMP-7	−2965.53	−2924.22	ASP6 ASP22 ASP27 GLU33 GLU70
**Pillar2a1b-HA-BMP-7**	**−4157.33**	**−4098.97**	**ASP6 ASP22 GLU15 GLU33 GLU70**
Pillar2a2b-HA-BMP-7	−3511.39	−3442.33	ASP6 ASP27 GLU15 GLU70 SER50 TYR51
Flat-HA-BMP-7-derived-peptide	−236.46	−241.04	GLN2 ASN4
Groove2a-HA-BMP-7-derived-peptide	−411.07	−408.40	LYS1 GLN2 ASN4
Concave2a1b-HA-BMP-7-derived-peptide	−1213.18	−1200.36	LYS1 ASN4 ASP12
Pillar2a1b-HA-BMP-7-derived-peptide	−918.35	−904.65	ASP12 ASP13 GLN2 LYS1
Flat-HA-BMP-7-no water	−7407.30	−7349.66	Many residues
Flat-HA-BMP-7-derived-peptide-no-water	−2861.26	−2825.55	Many residues

From the results of MD simulations, DFT based on the quantum mechanics of the Flat-HA-BMP-7/Flat-HA-BMP-7-derived peptide models was used to investigate atomic charges between the main adsorption residues of proteins and Ca atoms of the flat HA surfaces using the Dmol3 program in Materials Studio (Accelrys, San Diego, CA, USA)^48^. During simulation, the DNP double numerical basis set was used, which is comparable to the 6–31 G** basis set. A DFT semi-core pseudo potential with double-numerical basis set plus d functions was utilized^49^. The exchange-correlation energy was determined using the Perdew–Burke–Ernzerhof generalized gradient approximation^50^. Integrations over reciprocal space were based on the Monkhorst-Pack scheme with a 2 × 2 × 1 k-point mesh. Fermi smearing was 0.005 Ha (1 Ha = 27.211 eV). All atoms in our systems were relaxed during simulation. A vacuum thickness of 25 Å was chosen along the c-direction to avoid interactions among the adjacent cells. Convergence in self-consistent field tolerance, energy tolerance, a maximum force tolerance, and a maximum displacement tolerance were optimized at 1.0 × 10^–6^ Ha/atom, 1.0 × 10^–5^ Ha/atom, 0.002 Ha∕ Å, and 0.005 Å, respectively.

## Results and Discussions

### Adsorption behaviors between BMP-7 and atomic-level nano-structured HA surfaces

**Figure 3:**
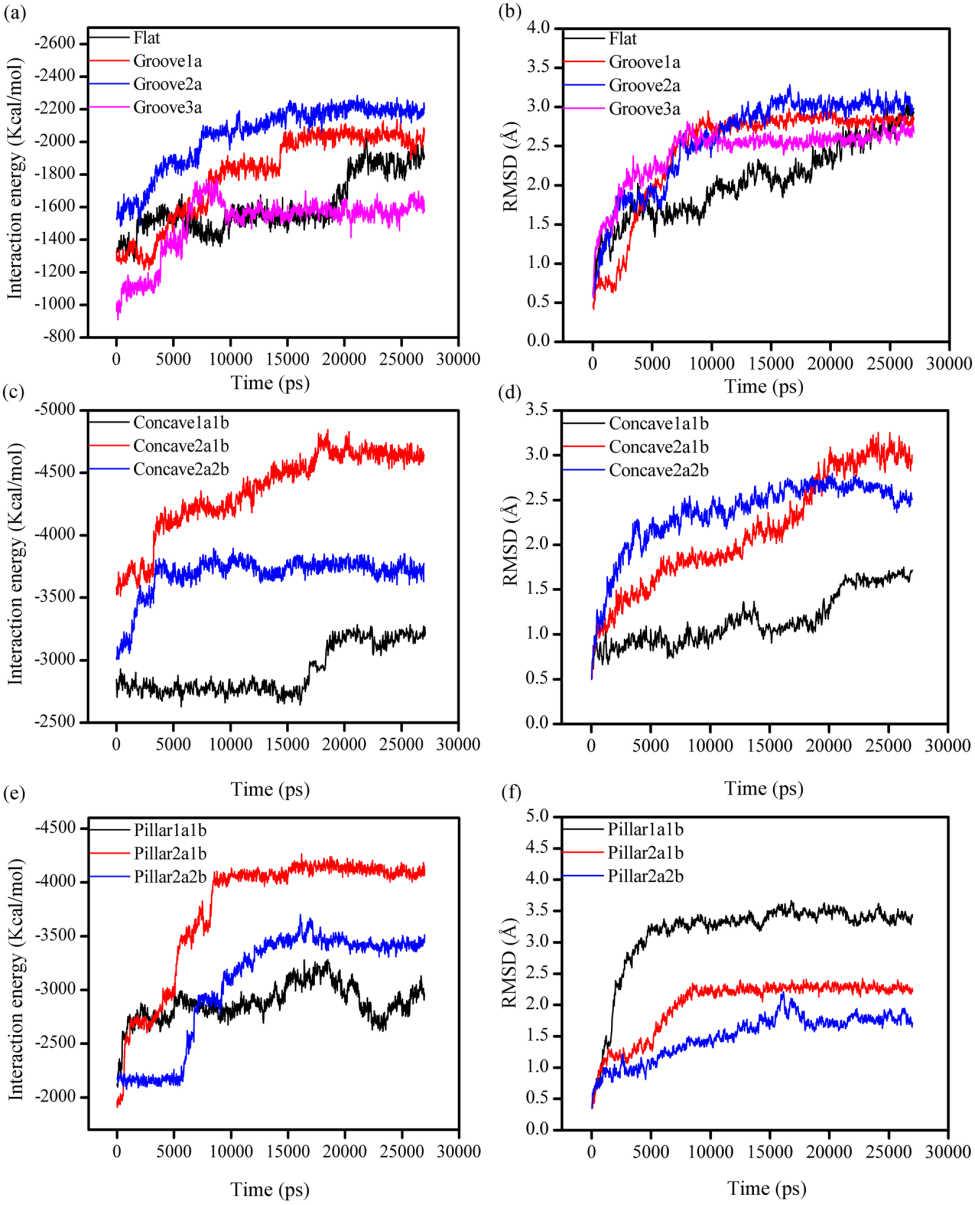
The interaction energy and RMSD between the BMP-7 and HA surfaces over the last 27000 ps simulation time: flat and groove HA surfaces, (**a**) and (**b**); concave HA surfaces, (**c**) and (**d**); pillar HA surfaces, (**e**) and (**f**).

The interaction energy and the root mean square deviation (RMSD) are usually adopted to evaluate whether the system has achieved stability or not^45,51^. The graph of interaction energy and RMSD versus simulation time is shown in Fig. 3. The results indicated that protein adsorption on various HA surfaces fluctuated mildly in the end of the simulation and had eventually achieved equilibrium.

**Figure 4:**
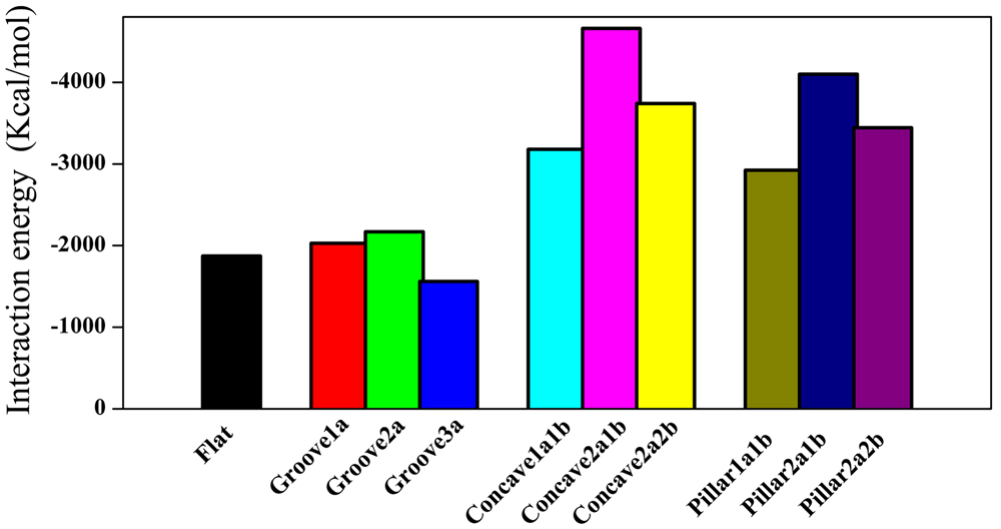
Average interaction energies between BMP-7 and flat, nano-groove, nano-concave and nano-pillar HA surfaces in the last 100 ps simulation time.

The adsorptions of BMP-7 on all nano-structured HA surfaces were thermodynamically favorable because the interaction energies were all negative, as shown in Table 2 and Fig. 4. The interaction energy was -1873.59 Kcal·mol^-1^ between BMP-7 and the flat HA surface. When BMP-7 interacted with nano-groove HA surfaces, the interaction energies indicated that adsorptions initially became strong and then became weak as the grooves of the HA surfaces gradually widened. Moreover, the interaction energy on the groove2a HA surface was 17.3% more negative than that on the flat HA surface. When BMP-7 was adsorbed on the nano-concave and nano-pillar HA surfaces, the same results were observed. However, the interaction energies suggested that there were stronger interactions between BMP-7 and nano-concave and nano-pillar HA surfaces than those between BMP-7 and flat or nano-groove HA surfaces. In particular, the interaction energies of BMP-7 on the concave2a1b and Pillar2a1b HA surfaces were nearly twice that of BMP-7 on the flat HA surface. These results demonstrated that the atomic-level nano-structured HA surfaces noticeably affected BMP-7 adsorption. Furthermore, nano-concave and nano-pillar HA surfaces greatly enhanced BMP-7 adsorption.

**Figure 5:**
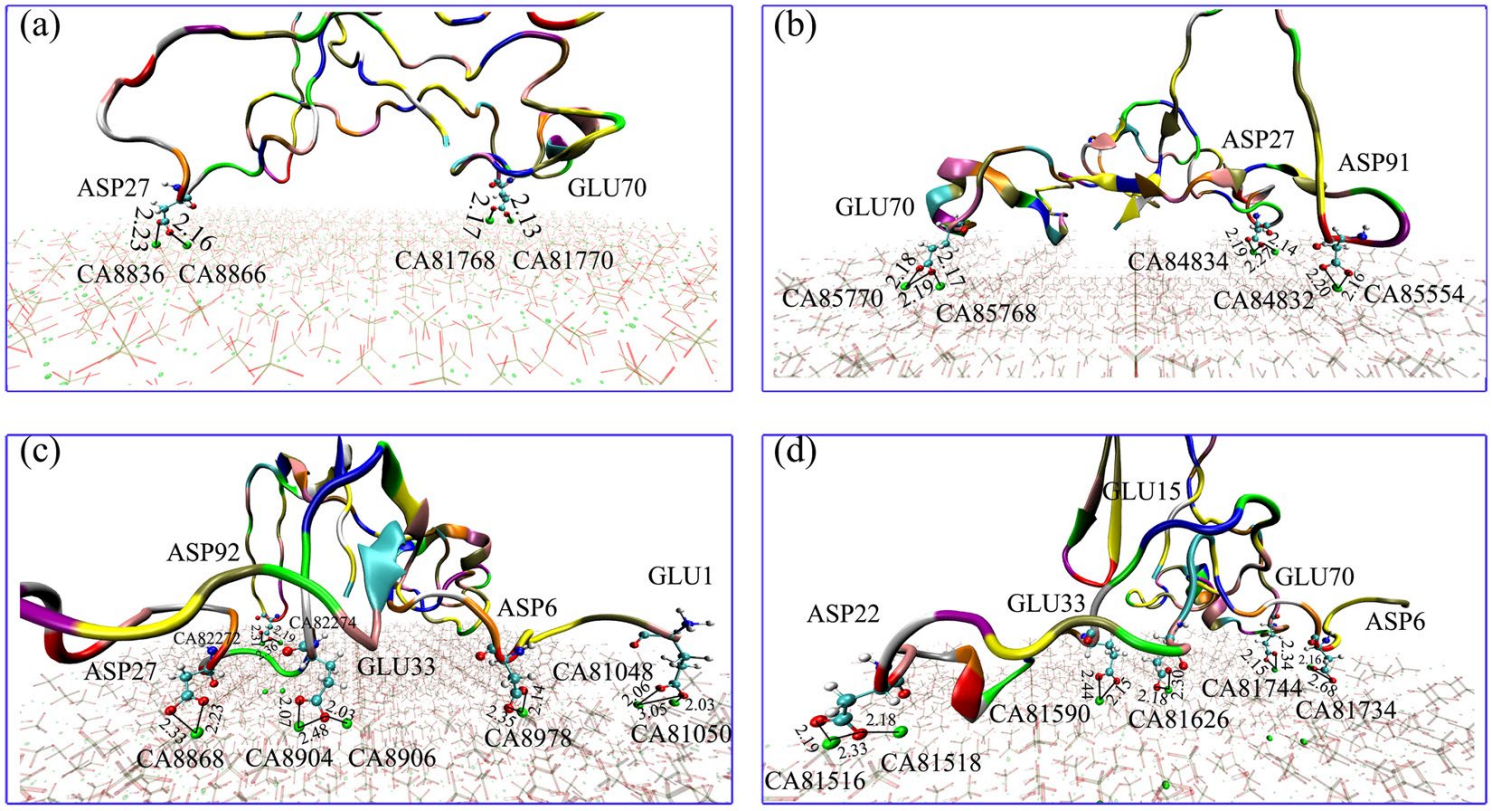
Interaction distances of BMP-7 adsorbing on the (**a**) flat, (**b**) groove2a, (**c**) concave2a1b, and (**d**) pillar2a1b HA surfaces after 30000 ps MD simulation. Water molecules are omitted for clarity.

The interactions between BMP-7 and HA mainly depended on electrostatic interaction (*E*_*elec*_) (Table 2). Based on the trajectory animation, BMP-7 adsorptions on different atomic-level HA morphology surfaces involved similar adsorption residues (Fig. 5). The primary interaction residues were ASP and GLU when BMP-7 interacted with the various HA surfaces (Fig. 1a). Both aspartic acid and glutamic acid residues are acidic amino acids and have one carboxyl group. Therefore, strong electrostatic interactions play an important role involving Ca atoms of HA and O atoms of carboxyl groups. Azzopardi *et al*.^52^ also indicated that the adsorption of the acidic protein osteopontin to Ca-rich HA (100) surfaces is regulated by electrostatic interactions. In general, the strength of such interactions is closely related to the interaction distance. Moreover, shorter interaction distances or greater numbers of interaction sites will result in larger interaction energies. The distances within 3 Å of Ca-O and H-bonds between BMP-7 and nano-structured HA surfaces are presented in Table 3 and Supplementary Table S1. The quantity and length of these distances indicated that their interactions were stronger on nano-groove, nano-concave, and nano-pillar HA surfaces (except groove 3a) than that on the flat HA surface. We also observed the strongest interaction to be between BMP-7 and the concave2a1b HA surface because of the shorter distances and greatest number of interaction sites within 3 Å (Fig. 5c). In addition, there were five O-Ca-O “bidentate” interactions between the O atoms of GLU1, GLU33, ASP6, ASP27, and ASP92 and Ca atoms of concave2a1b HA surfaces. When the number of interaction distances within 3 Å of BMP-7 on the groove3a HA surfaces reached its lowest value, the interaction energy also exhibited its lowest value (Supplementary Table S1). These results are comparable to those previous investigations of interactions between biomolecules and biomaterials. Wang *et al*.^53^ found that the adsorption of Gly on graphene oxide (GO) is stronger than that on graphene (G) because the H-bond distance between the Gly and GO is smaller than that from Gly to the center of G.

**Table 3:** Distances of Ca-O interaction or H-bonds between BMP-7 and flat, nano-groove, nano-concave, and nano-pillar HA surfaces. The unit of distance is Å.

Models	Distances (Å)	Models	Distances (Å)
Flat-HA-BMP-7	ASP27:OD2-CA8866:CAL = 2.18	Groove2a-HA-BMP-7	GLU70:OE2-CA85770 = 2.18
			GLU70:OE1-CA85770 = 2.19
	ASP27:OD1-CA8836:CAL = 2.23		GLU70:OE1-CA85768 = 2.17
	GLU70:OE2-CA81768:CAL = 2.17		ASP27:OD2-CA84834 = 2.19
	GLU70:OE1-CA81770:CAL = 2.13		
			ASP27:OD1-CA84834 = 2.27
			ASP27:OD1-CA84832 = 2.14
			ASP91:OD2-CA85554 = 2.20
			ASP91:OD1-CA85554 = 2.16
Concave2a1b-HA-BMP-7	GLU1:OE1-CA81050 = 2.03	Pillar2a1b-HA-BMP-7	GLU15:OE1-CA81626 = 2.30
	GLU1:OE1-CA81048 = 3.05		GLU15:OE2-CA81626 = 2.18
	GLU1:OE2-CA81048 = 2.06		GLU33:OE1-CA81590 = 2.44
	GLU33:OE1-CA8906 = 2.03		GLU33:OE2-CA81590 = 2.15
	GLU33:OE1-CA8904 = 2.48		GLU70:OE2-CA81744 = 2.34
	GLU33:OE2-CA8904 = 2.07		GLU70:OE1-CA81744 = 2.15
	ASP6:OD1-CA8978 = 2.14		ASP6:OD1-CA81734 = 2.16
	ASP6:OD2-CA8978 = 2.35		ASP6:OD2-CA81734 = 2.68
	ASP27:OD2-CA8868 = 2.33		ASP22:OD2-CA81518 = 2.18
	ASP27:OD1-CA8868 = 2.23		ASP22:OD1-CA81516 = 2.19
	ASP92:OD1-CA82274 = 2.19		ASP22:OD2-CA81516 = 2.33
	ASP92:OD1-CA82272 = 2.36		
	ASP92:OD2-CA82272 = 2.31		

**Figure 6:**
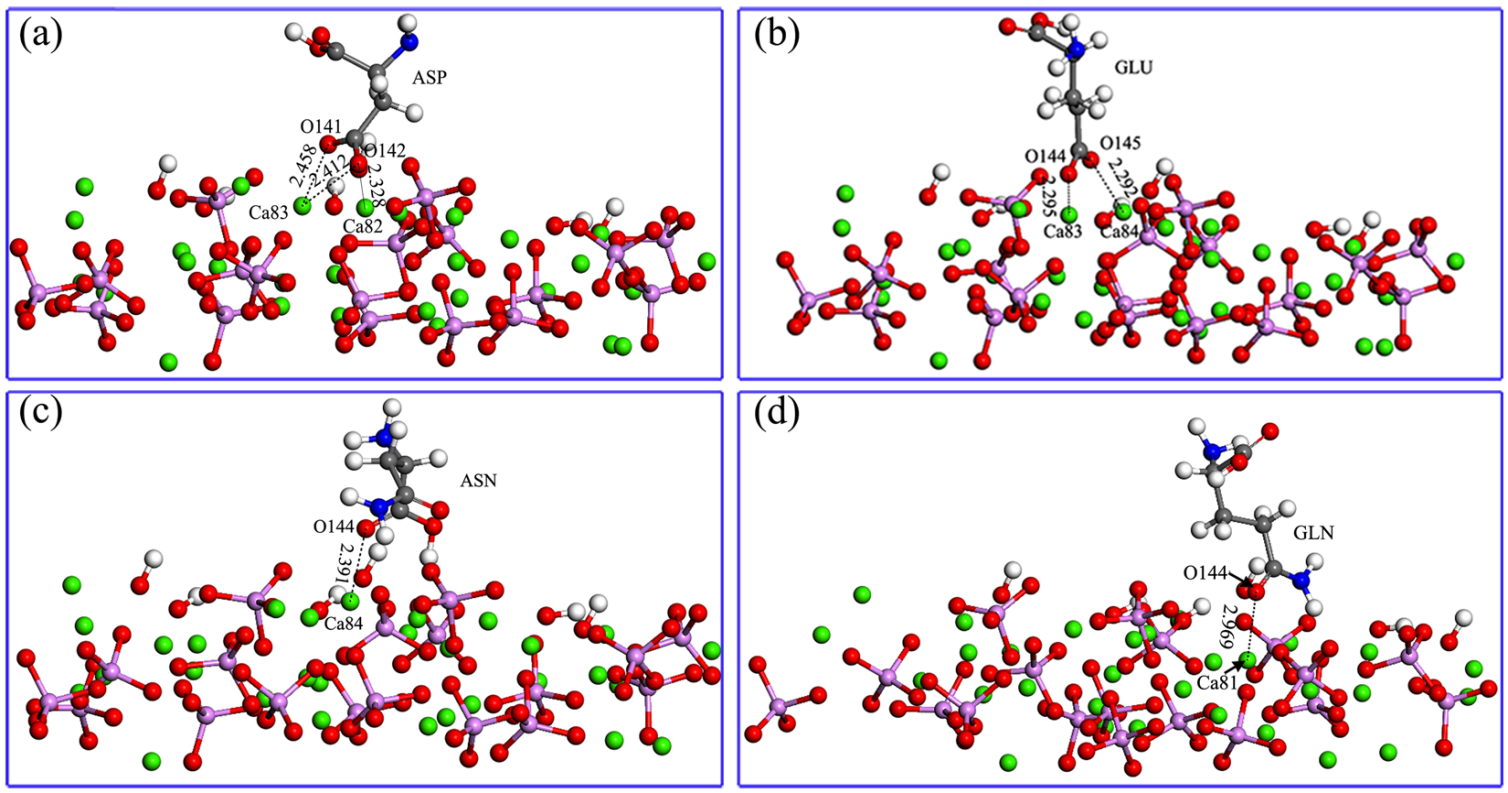
(**a**) and (**b**) ASP and GLU of BMP-7 and (**c**) and (**d**) ASN and GLN of BMP-7-derived peptide interacting with flat HA surfaces.

The DFT method is particularly appropriate for investigating physical properties based on electron density by measuring changes of atomic charges^54^. Electrostatic interactions between BMP-7 and all HA surfaces were dominant in this study. Thus, the atomic charges of the main adsorbed residues on flat HA surfaces changed before and after adsorption. The charge distributions of O atoms of –COO– in ASP27 and GLU70 and Ca atoms of HA were evaluated. The atomic charges of O atoms increased and atomic charges of Ca atoms decreased (Table 4), which indicated that the charge transfer mainly occurred from Ca atoms in HA to O atoms of –COO^−^. Furthermore, the distances between the O atoms of –COO^−^ and Ca atoms were 2.458, 2.412, 2.328, 2.295, and 2.292 Å, which are typical of O-Ca-O “bidentate” or Ca-O “monodentate” electrostatic interactions (Fig. 6a and b). The DFT results are in accordance with the above MD conclusions.

**Table 4:** Change in atomic charge of BMP-7 and its derived peptide on flat HA surfaces.

	Flat-HA-BMP-7					Flat-HA-BMP-7-derived peptide			
	atoms	Before adsorption	After adsorption	△e		atoms	Before adsorption	After adsorption	△e
ASP27	Ca82	1.524	1.538	0.014	ASN4	Ca84	1.524	1.564	0.040
	Ca83	1.558	1.561	0.003		O144	−0.463	−0.605	−0.142
	O141	−0.362	−0.614	−0.252		O145	−0.375	−0.388	−0.013
	O142	−0.340	−0.757	−0.417					
GLU70	Ca83	1.541	1.566	0.025	GLN2	Ca84	1.539	1.542	0.003
	Ca84	1.558	1.580	0.022		O144	−0.463	−0.514	−0.051
	O144	−0.356	−0.629	−0.273					
	O145	−0.356	−0.664	−0.417

**Figure 7:**
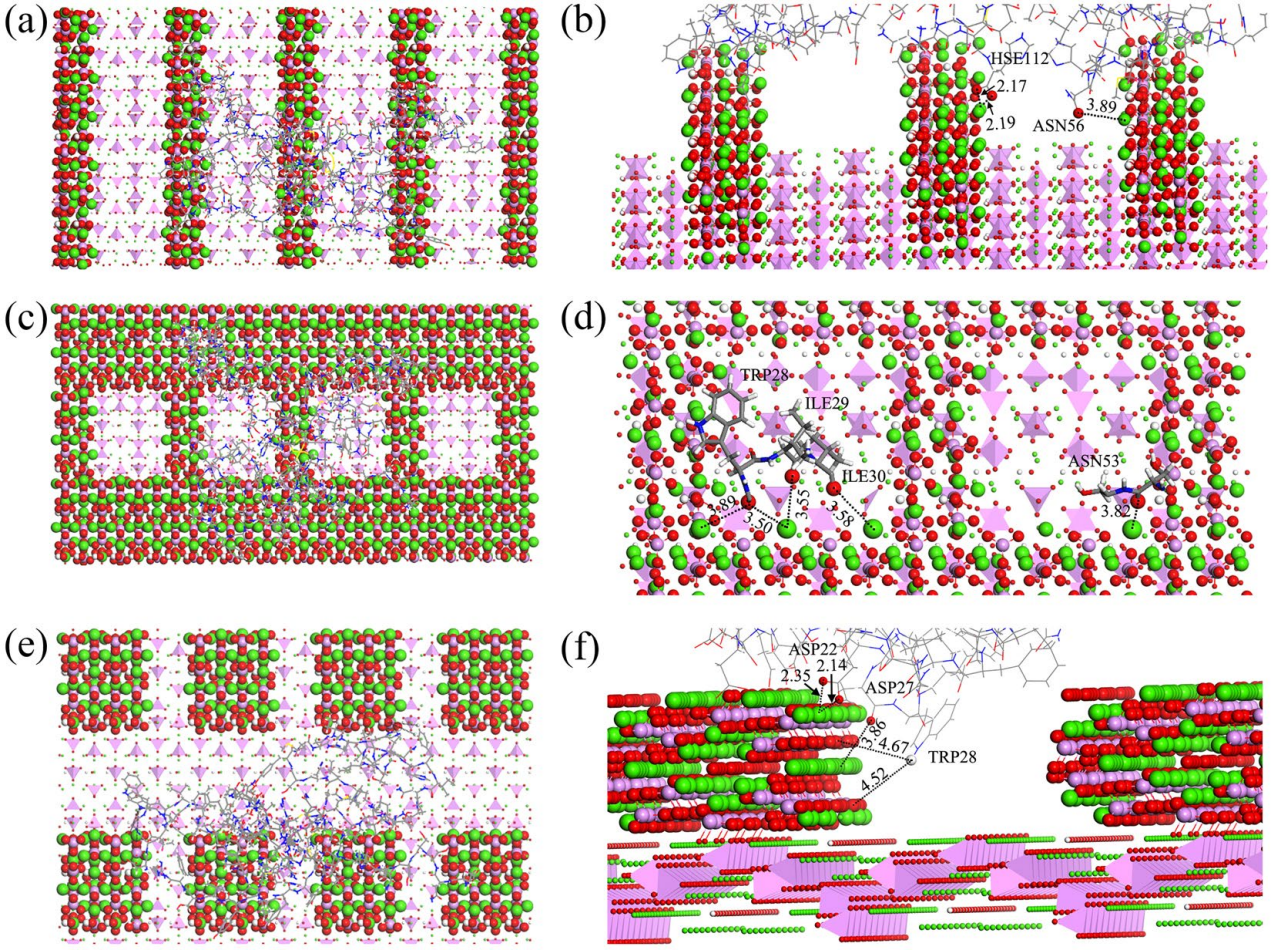
Residues of BMP-7 adsorbing on the (**a**) and (**b**) groove2a, (**c**) and (**d**) concave2a1b, (**e**) and (**f**) pillar2a1b HA (110) side surfaces after 30000 ps MD simulation, side and top views, respectively. Water molecules are omitted for clarity.

Variation in adsorption behavior of BMP-7 may be related to the HA surface morphology and to additional surfaces (HA (001) or HA ($1\bar{1}0$)). The implementation of atomic-level morphology may be achieved by increasing the surface area. In this study, the changes in the surface areas of the nano-groove, nano-concave, and nano-pillar HA surfaces are shown in Table 1. Although all surface areas increased compared to that of the flat HA surface, the change in surface area was disproportionate to the change in the interaction energy, suggesting that an increased surface area was not the only factor affecting interaction energies. The extent of the size matches, such as between groove and protein residues, also plays a crucial role in the interactions. For the groove2a-HA surface, in addition to ASP27, ASP91 and GLU70 absorbed Ca atoms of the HA surface within 3 Å (Fig. 5b), the O atoms of ASN56 and HSE112 residues fell into the grooves and interacted with the Ca atoms of HA (001) surface (Fig. 7b). This may be because ASN56 and HSE112 were approximately 6.816 and 7.600 Å in size, respectively, whereas the width of goove2a was 13.758 Å. Thus, ASN56 and HSE112 can spread into the groove. For the concave2a1b-HA surface, the TRP28, ILE29, ILE30, and ASN53 residues fell into the concaves and interacted with the Ca atoms of the HA (001) surface (Fig. 7d). For the Pillar2a1b-HA surface, ASP22, ASP27, and TRP28 residues could firmly bind the HA (001) surfaces (Fig. 7f). The sizes of TRP28, ILE29, and ASP22 were approximately 8.565, 6.285, and 5.351 Å, respectively. These sizes allowed them to spread into the nanopore and interact with the HA (001) or (1$\bar{1}$0) surface.

There were clearly not only more interaction sites between BMP-7 and atomic-level nano-structured HA surfaces than those between BMP-7 and the flat HA surface, but also the sizes of several residues matched the lengths and widths of the nanopores in the nano-groove, nano-concave and nano-pillar HA surfaces. Therefore, the atomic-level HA morphology significantly affected BMP-7 adsorption. These results may be confirmed by experimental studies. For example, Coata *et al*.^55^ and Mao *et al*.^56^ have shown that the surface morphology of HA has obvious effects on the adhesion, proliferation, and differentiation of cells.

### Adsorption behaviors between BMP-7-derived peptide and atomic-level nano-structured HA surfaces

The interaction behaviors between BMP-7**-**derived peptide (KQLNALSVLYFDD) and the flat, nano-groove2a, nano-concave2a1b, and nano-pillar2a1b HA surfaces were studied. Interactions energies between the peptide and various HA surfaces are shown in Table 2. The interaction energy was -241.04 Kcal/mol when BMP-7-derived peptide interacted with the flat HA surface, and two residues (ASN4 and GLN2) bound the HA surfaces tightly. Interaction distances between O atoms of ASN4 and GLN2 and Ca atoms of HA were 2.21, 2.18, and 2.13 Å, respectively. The interaction increased when BMP-7-derived peptide was adsorbed onto the nano-groove2a HA surface (-408.40 Kcal/mol), and three residues (LYS1, ASP12, and ASP13) were firmly absorbed onto the HA surface. The energy further decreased when the derived peptide was absorbed onto the nano-concave2a1b and nano-pillar2a1b HA surfaces (−1200.36 Kcal/mol and -904.65 Kcal/mol). These results showed that there were stronger adsorptions of BMP-7-derived peptide on the nano-groove, nano-concave, and nano-pillar HA surfaces than that on the flat HA surface. The strongest adsorption was observed for BMP-7-derived peptide on the nano-concave2a1b HA surface.

**Figure 8:**
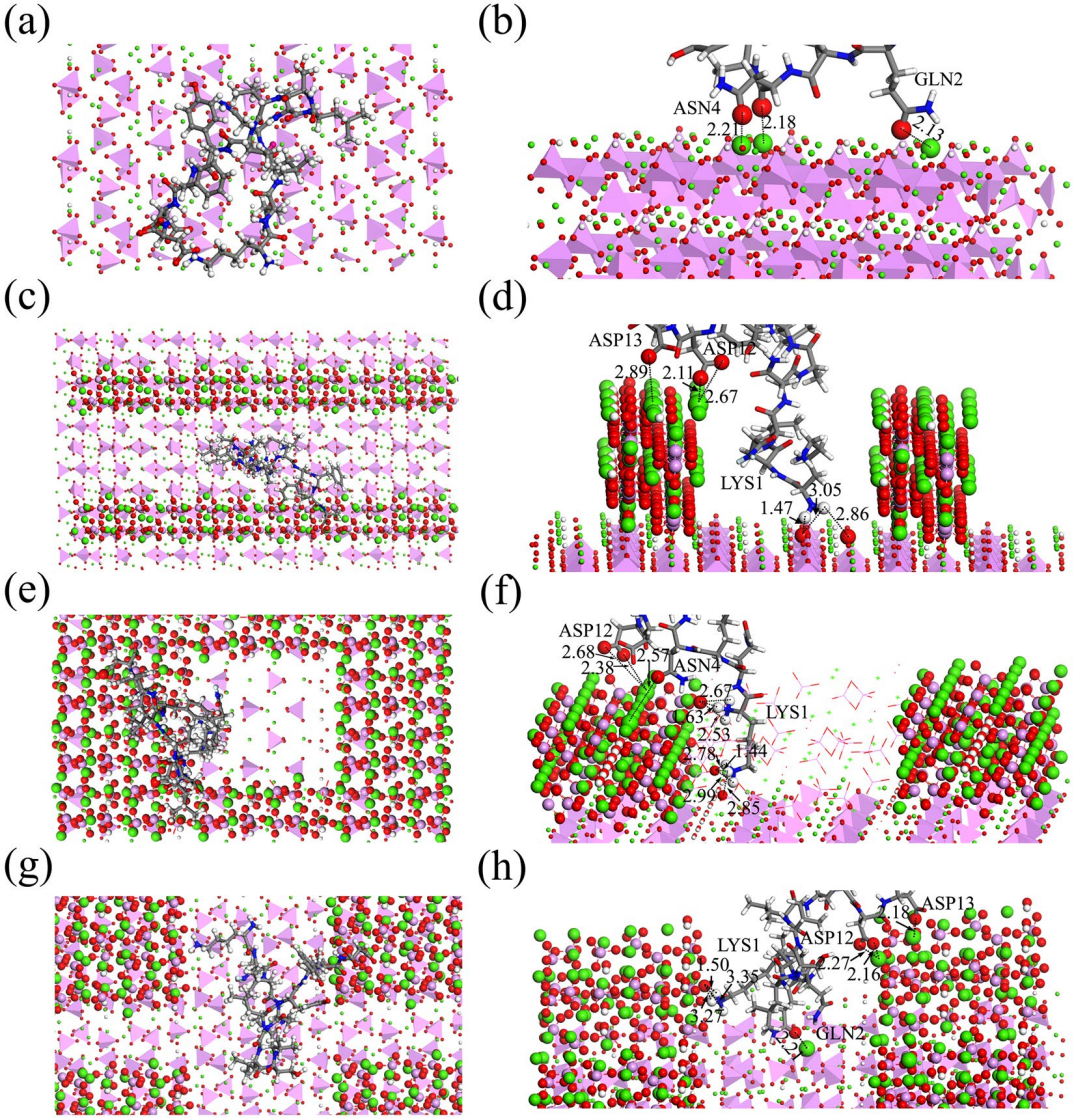
Residues of BMP-7-derived peptide adsorbing on the (**a**) and (**b**) flat, (**c**) and (**d**) groove2a, (**e**) and (**f**) concave2a1b, (**g**) and (**h**) pillar2a1b HA surfaces after 30000 ps MD simulation, side and top views, respectively. Water molecules are omitted for clarity.

Furthermore, we found that the KQLNALSVLYFDD peptide expanded slowly on the nano-groove, nano-concave, and nano-pillar HA surfaces with an increase in adsorption time, and there were more carboxyl or amino groups interacting with the Ca atoms, phosphate and hydroxyl groups of HA surfaces, as shown in Fig. 8. Electrostatic interactions remained dominant (Table 2). The charge transfer of the –COO^−^and ketone group of ASN4 and GLN2 in the derived peptide and Ca atoms of HA were also investigated by the DFT method, and similar simulation results with BMP-7 adsorption on the flat HA surface were obtained (Fig. 6c and d, and Table 4). Figure 6c and d showed that there were Ca-O electrostatic interactions between the O atoms of ASN4 and GLN2 and Ca atoms in HA. The data presented in Table 4 clearly indicate that O atoms of ASN4 and GLN2 acquired electrons and Ca atoms lost electrons, forming Ca-O electrostatic interactions. Therefore, electrostatic interactions between the derived peptide and HA are dominant, which is consistent with reported interactions between other peptides and HA surfaces. For example, Almora-barrios *et al*.^57^ employed DFT to investigate the interaction of HA and PRO- hydroxyproline (HYP)-GLY, HYP-PRO-GLY, PRO-LYS-GLY, and PRO-hydroxylysine (HYL)-GLY, and discovered that Ca–O electrostatic interactions prevail between O atoms of the carboxyl and Ca atoms of HA.

Alteration trends of interaction energies between the BMP-7-derived peptide and nano-structured HA were similar to those between BMP-7 and nano-structured HA. Thus, nano-groove, nano-concave and nano-pillar HA surfaces largely enhanced BMP-7-derived peptide adsorption. Additionally, we found that the active sites of BMP-7-derived peptide (a small molecule) may easily spread out and stretch inside the trenches to absorb firmly on the side surfaces of nano-groove, nano-concave, and nano-pillar HA, such as the HA (001) surface or HA (1$\bar{1}$0) surface, as illustrated in Fig. 8. In the groove2a-HA model, the LYS1 residue fell into the bottom of the groove; we observed interactions between the amino group of LYS1 and O atoms of the hydroxyl group of the HA (110) surface (Fig. 8d). In the concave2a1b-HA model, the LYS1 residue slipped into the groove and possibly pulled the ASP12 and ASN4 close to the nano-concave2a1b HA surfaces. There were Ca-O interactions between the O atoms of ASP12 and ASN4 and Ca atoms of the HA (110) surface. Additionally, the LYS1 residue was surrounded by the HA (001) and (1$\bar{1}$0) surfaces; thus, the H atoms of LYS1 interacted with the O atoms of -PO_4_ and -OH on the HA (001) surface (Fig. 8f). In the pillar2a1b-HA models, both the LYS1 and GLN2 residues contacted the HA (001) and (110) surfaces upon entering the groove of the nano-pillar HA surface, and the H atoms of LYS1 or O atoms of GLN2 interacted with the O atoms of -PO_4_ in HA (001) or Ca atoms of the HA (110) surface, respectively (Fig. 8h).

The sizes of LYS1 and GLN2 are approximately 9.40 and 6.67 Å, respectively, which completely match the width of the grooves (13.758 Å) in goove2a, concave2a1b, and pillar2a1b HA models. These phenomena also demonstrated that the atomic-level surface morphology of HA is pivotal for peptide adsorption. Thus, a compatible groove size of surface morphology would be beneficial for promoting specific peptide adsorption. These results are consistent with those of Zhang *et al*.^58^, who found that the interaction was strengthened when the RGD peptide fell into the grooves between the step edges of TiO_2_.

### Water Effects

The effects of water on BMP-7 and its derived peptide adsorptions on flat HA surfaces were also considered by MD and DFT studies. The MD conclusions suggested that the water layer inhibited BMP-7 and its derived peptide absorbing on flat HA surfaces quickly and steadily through intermolecular H-bonds between the H_2_O and HA or between the H_2_O and protein (Supplementary Fig. S5). The DFT results suggested that “–COO^–^…H_2_O…OPO_3_” water-bridged H-bonds between the anionic Asp molecule, H_2_O molecule and PO_4_ in HA and H-bonds between the -NH_2_ and H_2_O molecule were formed, moreover, the water molecules hindered the main “Ca-O” interaction between the ASP and Ca atoms in HA (Supplementary Fig. S6d and f). These results are in accordance with previous literature^59,60^. Wang *et al*.^45^ also reported that water molecules on HA (110) surfaces could prevent BSA protein from migrating close to the surface. Furthermore, there are water-bridged H-bonds between amino acid residues, H_2_O and HA substrate. The MD and DFT conclusions confirmed that water environments seriously affected the adsorption of amino acids on HA (110) surfaces. Details are presented in the Supplementary Information.

### Consideration of other influencing factors

It should be noted that, except for the micro-nano structured surface morphology, other influencing factors should also be considered during MD simulations, including the effects of pH, ionic strength, and surface chemistry, which renders the simulation and analysis of protein structures more complicated^10^. Researchers suggest that the change of pH, ionic strength, surface chemistry and other factors will eventually result in changes to surface charge intensity and distribution, and further affect the interaction between proteins and HA. For example, when the change of pH or ionic strength causes the Zeta-potential value to decrease, most proteins are prone to adsorbing on HA surfaces due to the decrease in electrostatic repulsions between the HA and the negatively charged protein^61^. Yin *et al*.^62^ found that pH increases resulted in a decreasing adsorption of BSA onto HA surfaces, and NaCl concentration increases caused the adsorption to increase. These effects are attributed to the difference in the negative Zeta-potential of HA being increased due to the pH increase, whereas increases in ionic strength resulted in a decreased negative Zeta-potential of HA. Furthermore, the HA surface environment can be altered by dopants, further affecting interactions with protein. Chen *et al*.^63^ investigated the adsorption and desorption behaviors of leucine-rich amelogenin protein (LRAP) on a series of SiHA (100) surfaces. They discovered that the Si doping into the HA (100) surface increases the surface negative charge due to induction of SiO_4_^4-^, which leads to the -NH_3_^+^, -NH_2_, and -OH moving towards the surface, and the negatively charged -COO^−^ being repelled from the surface. Therefore, the interaction of –COO^−^ and Ca^2+^ becomes gradually weaker due to the effects of charge rejection and steric hindrance of large-sized SiO_4_^4-^ molecules.

Much work involving these factors has been reported, whereas a consensus on which are critical factors has not been reached so far. The interaction behaviors between materials and organic molecules are often the result of the joint actions of different factors. In this study, we mainly considered the effects of different nanoscale surface morphologies of HA substrates on the adsorption of proteins, which is rarely reported. Our results revealed that surface nano-morphology is also an important factor that affects the interaction between HA and protein, in addition to previously-reported factors. In addition, we implemented an abstract model that represents those nano-morphology materials used in previous experimental studies. Although the conclusions drawn from computer simulations cannot fully reflect experimental results, they can still provide meaningful explanations for experimental phenomena within a limited simulation scale and offer possible theoretical guidance for further study on the design of biological nanomaterials.

## Conclusions

To explore the effects of different surface morphologies of HA materials on the adsorption of biomolecules, we elucidated the interaction effects of BMP-7 and BMP-7-derived peptide on flat, nano-groove, nano-concave and nano-pillar HA (110) surfaces using MD and DFT methods. Our results indicated that the atomic-level morphology of HA significantly affects the interaction strength between protein/peptide and HA substrates. The interactions of BMP-7 and its derived peptide with the nano-concave and nano-pillar HA surfaces were stronger than those with flat and nano-groove HA surfaces. At the same time, the results also revealed that if the groove size of the nano-structured HA surfaces matches that of residues in the protein or peptide, the residues are likely to spread into the grooves in nano-groove, nano-concave, and nano-pillar HA, further strengthening the interactions. Our results improve the understanding of the adsorption behaviors of proteins on nano-structured HA surfaces. In addition, our results provide valuable theoretical guidance for designing new types of bioceramic materials useful for bone regeneration and tissue engineering applications.

## Electronic supplementary material

Supplementary Information
