# Supplementary material for: Effects of atomic-level nano-structured hydroxyapatite on adsorption of bone morphogenetic protein-7 and its derived peptide by computer simulation
Source: Sci Rep. 2017 Nov 9;7:15152. doi: 10.1038/s41598-017-15219-6 (PMC5680243; doi:10.1038/s41598-017-15219-6)
Supplement: Supplementary file 1 — Supplementary Information [file 41598_2017_15219_MOESM1_ESM.pdf]

Effects of atomic-level nano-structured hydroxyapatite on adsorption of bone morphogenetic protein-7 and its derived peptide by computer simulation

Qun Wang [a, b], Menghao Wang [a], Xiong Lu [a], Kefeng Wang [*, c], Liming Fang [d], Fuzeng Ren [e], Guoming Lu [f],

[a] Key Lab of Advanced Technologies of Materials, Ministry of Education, School of Materials Science and Engineering, Southwest Jiaotong University, Chengdu 610031, Sichuan, China

[b] College of Life Science and Biotechnology, Mian Yang Teachers' College, Mianyang 621006, Sichuan, China

[c] National Engineering Research Center for Biomaterials, Genome Research Center for Biomaterials, Sichuan University, Chengdu 610065, Sichuan, China

[d] Department of Polymer Science and Engineering, School of Materials Science and Engineering, South China University of Technology, Guangzhou 510641, China

[e] Department of Materials Science and Engineering, South University of Science and Technology of China, Shenzhen, Guangdong 518055, China

[f] School of Computer Science and Engineering, University of Electronic Science and Technology of China, Chengdu 610054, Sichuan, China

[*]Corresponding author. Tel: +86-28-85415030

Fax: +86-28-85415030

E-mail: fencal@163.com

Supplementary Table S1. Distances of Ca-O and H-bonds between the BMP-7 and flat, nano-groove, nano-concave and nano-pillar HA surfaces. The unit of distance is Å.

Models	Distances (Å)	Models	Distances (Å)	Models	Distances (Å)
Groove1a<br>-HA-BMP-7	GLU70:OE2-CA85808=2.08<br>GLU70:OE1-CA85808=2.28<br>GLU70:OE1-CA85802=2.21<br>ASP27:OD2-CA84870=2.19<br>ASP27:OD1-CA84870=2.12<br>ASP27:OD1-CA84868=2.13	Concave1a1b<br>-HA-BMP-7	ASP27:OD2-CA81592=2.22<br>ASP27:OD1-CA81592=2.16<br>ASP27:OD1-CA81586=2.21<br>ASP92:OD2-CA82272=2.07<br>ASP92:OD1-CA82274=2.31<br>ILE30:HD2-HYD1606:O=3.21<br>ILE30:HD2-HYD1608:O=2.89<br>ILE30:HB-HYD1608:O=3.18	Pillar1a1b<br>-HA-BMP-7	GLU70:OE2-CA81770=2.15<br>GLU70:OE2-CA81780=3.21<br>GLU70:OE1-CA81780=2.21<br>ASP6:OD1-CA81770=2.12<br>ASP6:OD2-CA81770=2.73<br>ASP22:OD2-CA81554=2.17<br>ASP22:OD1-CA81554=2.16<br>ASP27:OD2-CA81564=2.26<br>ASP27:OD1-CA81564=2.35
Groove3a<br>-HA-BMP-7	Glu70:OE2-CA85734=2.13<br>Asp27:OD1-CA84870=2.14<br>Asp91:OD2-CA85590=2.28<br>Asp91:OD1-CA85590=2.22	Concave2a2b<br>-HA-BMP-7	Glu15:OE1-CA81660=2.12<br>Glu15:OE2-CA81664=2.18<br>Asp6:OD1-CA81700=2.06<br>Asp6:OD2-CA81700=2.43<br>Asp27:OD2-CA81552=2.13<br>Asp27:OD2-CA81552=3.17<br>Asp27:OD1-CA81518=2.08	Pillar 2a2b<br>-HA-BMP-7	Glu15:OE2-CA8906=2.26<br>Glu15:OE1-CA8916=2.16<br>Glu15:OE2-CA8904=2.09<br>Glu70:OE1-CA81014=2.44<br>Glu70:OE2-CA81024=2.16<br>Glu70:OE1-CA81012=2.10<br>Asp6:OD1-CA8978=2.18<br>Asp6:OD2-CA8978=2.38<br>Asp27:OD1-CA8798=2.39<br>Asp27:OD2-CA8808=2.12

Adsorption behaviors of BMP-7 on HA surfaces with different termination

Different surface termination is critical for the interaction of proteins and biomaterials, because different termination not only determines the surface charge, but also the surface atomic structure and composition. In order to compare the effects of surface termination, we built two further models with different terminations according to the literature [1, 2], other than the manuscript HA (110) surfaces shown in Supplementary Fig. S1a. These three models were built with different ion distributions, named as Flat1 (Flat1 is the manuscript model, the number 1 is for the sake of distinguishing with 2 and 3. Note that in the other parts of the article, the Flat hydroxyapatite models

are all represented by Flat), Flat2 and Flat 3, as shown in Supplementary Fig. S1.

The Mullikan charges of all atoms in the different termination models are displayed in Supplementary Table S2, which was calculated by the DFT method. The MD simulation between BMP-7 and two Flat2 and Flat3 HA (110) surfaces were further calculated. The calculation parameters are consistent with those of the manuscript, and the MD simulation time was also 30000 ps. A graph of interaction energy and RMSD versus 27000 ps simulation time is shown in Supplementary Fig. S2. The results indicated that BMP-7 adsorption on the various HA surfaces fluctuated mildly at the end of the simulation and eventually achieved equilibrium states.

In the Flat2 and Flat3 models, the average interaction energies in the last 100 ps were -1525.24 and -1464.43 Kcal/mol, respectively (Supplementary Table S2). These interaction energies are approximately 300 Kcal/mol higher in the Flat2 and Flat3 models than that in the Flat1 model. This is because the charges of Ca atoms in the Flat2 and Flat3 models were lower than that in the Flat1 model, according to Supplementary Table S2.

The main absorbed residues of Flat2 and Flat3 (Supplementary Figs. S3b and S3c) are all ASP and GLU residues, which is similar to that in the Flat1 model (Supplementary Fig. S3a). The results indicated that in any termination condition, the O atoms of the protein tend to interact with the Ca atoms of the HA substrates. The interaction distances within 3 Å of Ca-O between BMP-7 and the various HA (110) surfaces are shown in Supplementary Fig. S3. In the Flat1 model, the O atoms of ASP27 and GLU70 interacted with the Ca atoms of the top layer, which formed four Ca-O interactions (Supplementary Fig. S3a). However, In the Flat2 and Flat3 models, the O atoms of ASP91, ASP92 and GLU70 interacted with the Ca atoms of the second layer, which also formed four Ca-O interactions (Supplementary Figs. S3b and S3c).

In summary, these results indicated that different termination of HA (110) surfaces

have little effect on the interaction between BMP-7 and HA. Although the atomic charges are slightly different, the interaction behaviors are similar. In fact, the surface of the solid HA is non-crystalline in the real world, which has different surface properties compared to the bulk ones. Unfortunately, there has been no well accepted model or force fields of non-crystalline HA at present. In order to simplify the calculations, all models were adopted as crystalline ones, which should make sense when they are compared at the horizontal level.

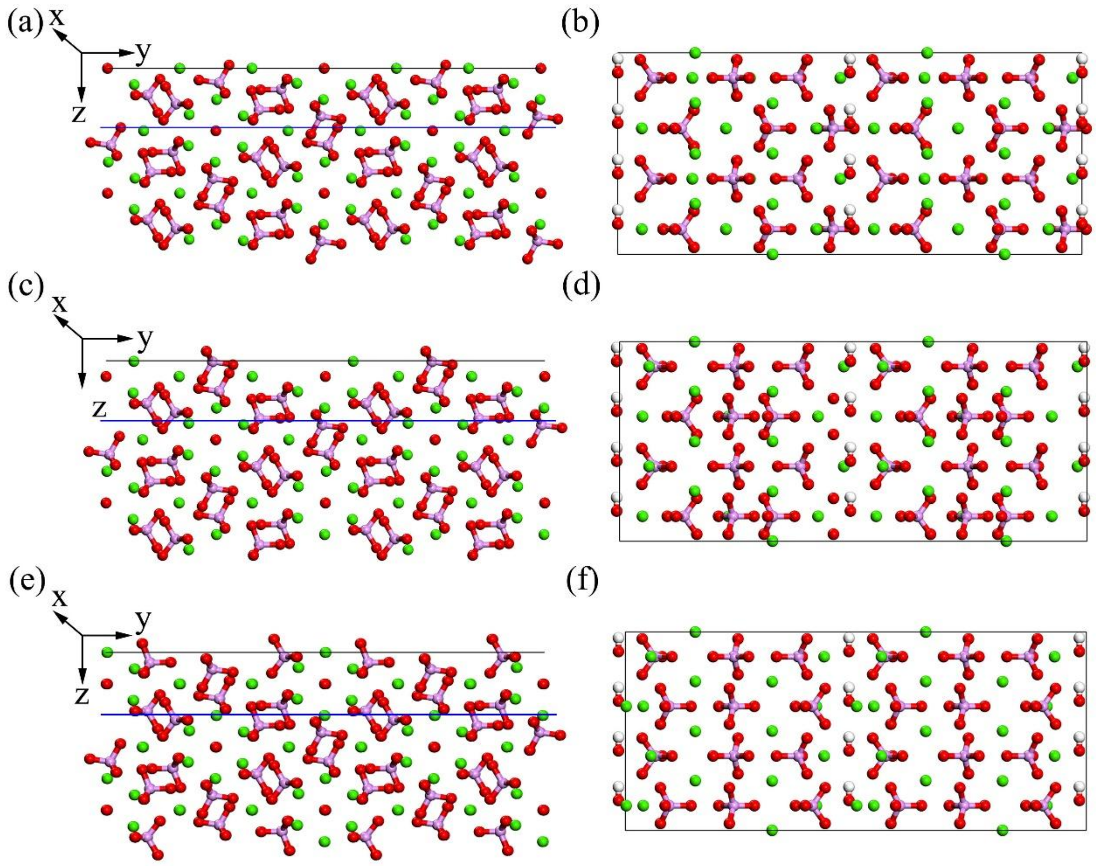

Supplementary Figure S1. (**a**) and (**b**) the termination of Flat1 model; (**c**) and (**d**) the termination of Flat2 model ; (**e**) and (**f**) the termination of Flat3 model, side and top views, respectively.

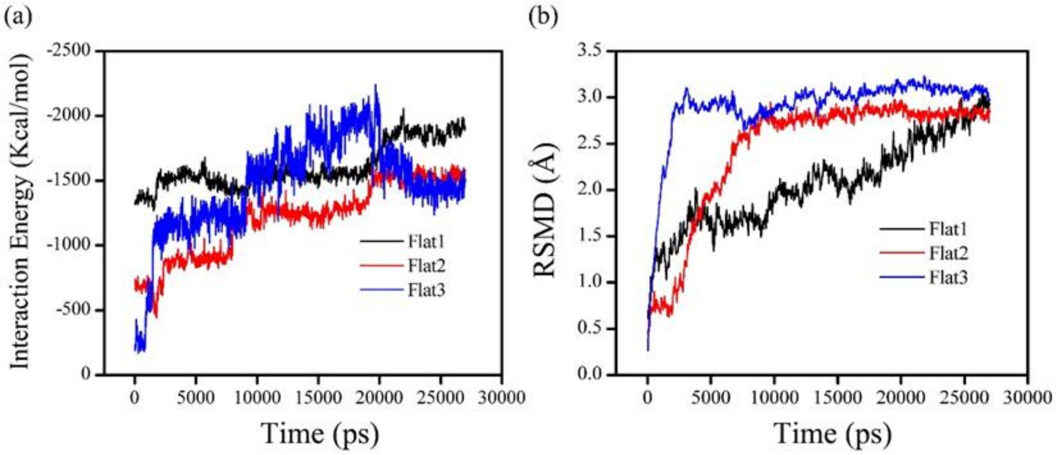

Supplementary Figure S2. Interaction energy and RMSD between BMP-7 and Flat1, Flat2, and Flat3 HA surfaces in the 27000 ps simulation time.

Supplementary Table S2. Atomic charges using the DFT method and average interaction energies of BMP-7 on the Flat1, Flat2 and Flat3 HA surfaces in the last 100 ps simulation time.

**Models**	**Ca**	**P**	**O(PO4)**	**H**	**O(OH)**
Flat1	2.00	2.60	-1.40	0.60	-1.60
Flat2	1.52	1.55	-0.96	0.28	-1.02
Flat3	1.52	1.51	-0.97	0.35	-0.84

**Models**	**ELEC Kcal·mol-1**	**Interaction energy Kcal·mol-1**	**Main absorbed residues**
Flat1	-1883.96	-1873.59	ASP27 GLU70
Flat2	-1500.72	-1525.24	ASP91 ASP92 GLU70
Flat3	-1466.12	-1464.43	ASP92 GLU70

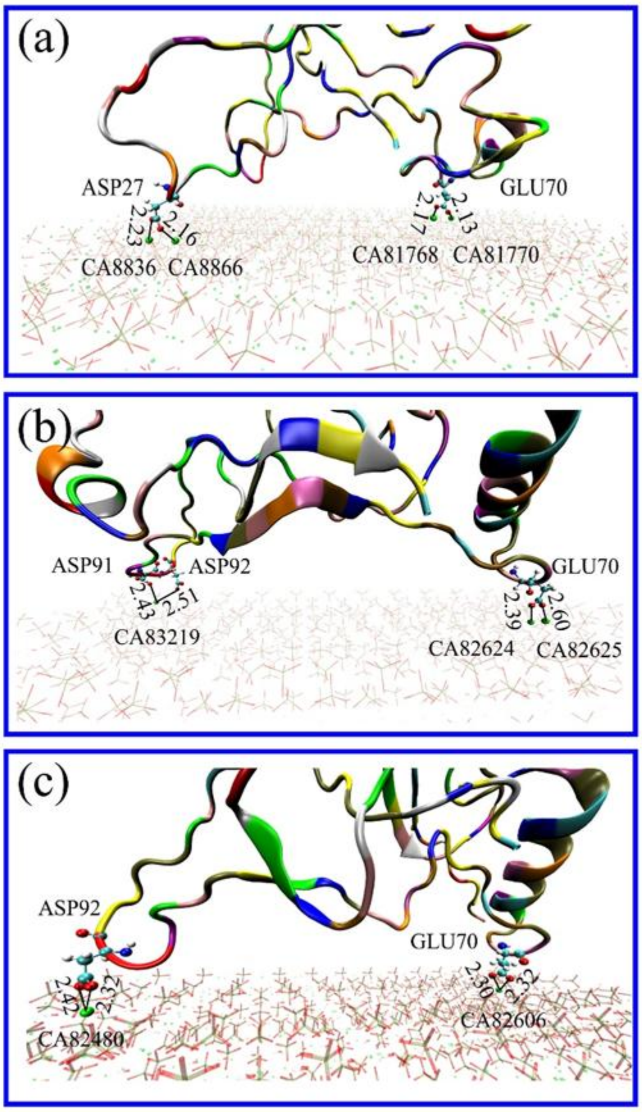

Supplementary Figure S3. Interaction distances of BMP-7 adsorbing on the (**a**) Flat1 surface; (**b**) Flat2 surface; (**c**) Flat3 surface after MD simulation. Water molecules are omitted for clarity.

Adsorption behaviors between BMP-7/BMP-7-derived peptide and Flat HA surfaces in the no-water system represented by MD simulation

We investigated the interactions of BMP-7 / BMP-7-derived peptide and Flat HA surfaces in the no-water system using the MD method (Supplementary Fig. S4). The interaction energy of BMP-7 on the flat HA surface in the no-water system (-7349.66

Kcal/mol) was more negative than that in the water system (-1873.59 Kcal/mol). There were as high as 46 residues strongly absorbing on the HA surface after approximately 500 ps MD simulation. Additionally, the interaction energy of BMP-7-derived peptide on the flat HA surface in the no-water system (-2825.55 Kcal/mol) was ten times lower than that in the water system (-241.04 Kcal/mol). Almost all residues of BMP-7 derived peptide were firmly adherent on the HA surface after MD simulation (Supplementary Fig. S4d). Furthermore, there was intermolecular hydrogen bonding (H-bonds) between H_2O and HA or between H_2O and protein/peptide in the water system (Supplementary Fig. S5), leading the water layer to inhibit BMP-7 and its derived peptide absorbing on flat HA surfaces.

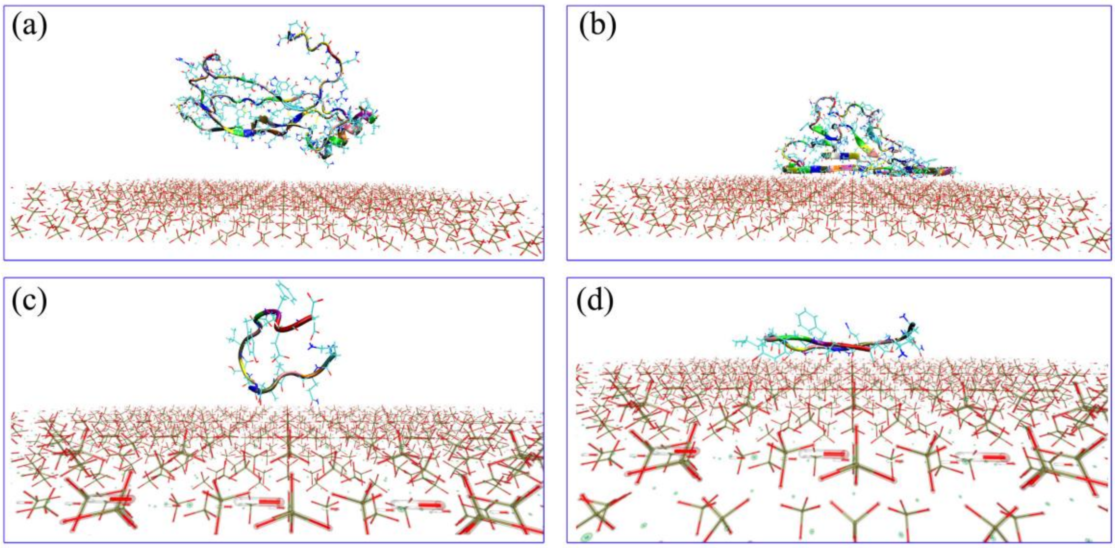

Supplementary Figure S4. Interactions of (**a**) and (**b**) BMP-7 and (**c**) and (**d**) BMP-derived peptide adsorbing on Flat HA surfaces before and after MD simulation. Note that a large number of residues adsorb on flat HA surfaces rapidly after the MD simulation.

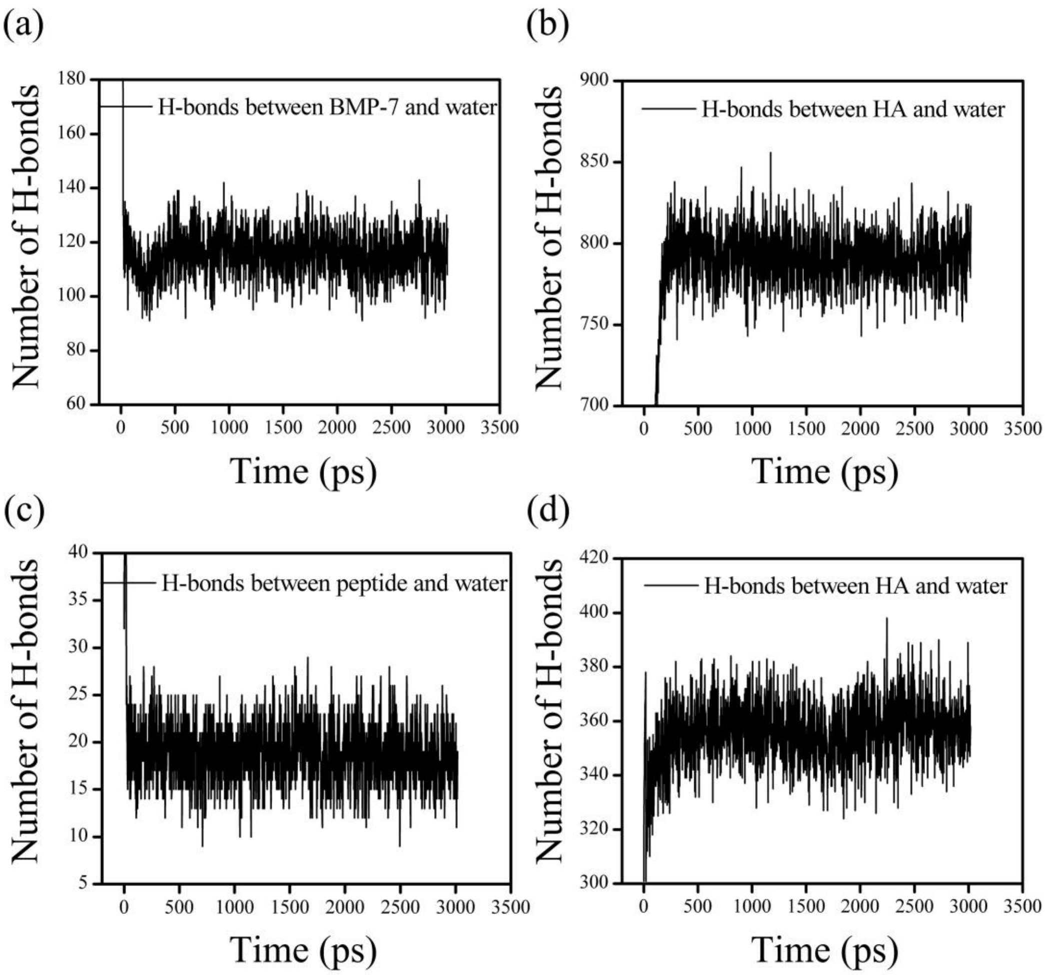

Supplementary Figure S5. Numbers of H-bonds (**a**) between BMP-7 and water and (**b**) between HA and water in the Flat-HA-BMP-7 system; (**c**) between the BMP-7-derived peptide and water and (**d**) between the HA and water in the Flat-HA-BMP-7-derived peptide system after 3000 ps simulation time.

Adsorption behaviors between BMP-7 and Flat HA surfaces in the water system by the DFT simulation

Proteins generally interact with biomaterial surfaces in aqueous solution or at solid-liquid interfaces [3, 4], therefore, the effect of water environment plays an important role in interaction between the protein and HA substrate. The amino acid

ASP, interacting with HA (110) surfaces in different water environments was investigated using the DFT method. ASP is one of the main residues of BMP-7, which plays a dominant role during the interaction of BMP-7 and HA. Two types of water models were built to study the solvent effect, a water environment model and a water layer model.

In the water environment model, water molecules surrounded the anionic ASP, termed the WE model (Supplementary Fig. S6c). The anionic ASP molecule was on the Flat HA (110) surface with the same configuration under vacuum (Supplementary Fig. S6a). Three water molecules were near the functional groups of anionic ASP molecule to saturate the deprotonated groups. Another three water molecules surrounded anionic ASP, representing a water environment. The interaction energy in the WE model was less than that in the vacuum model (Supplementary Table S3). Three "$-COO^{-}\cdots H_2O\cdots OPO_3$" water-bridged H-bonds were formed between the anionic ASP molecule, H_2O molecule and PO_4 in HA (Supplementary Fig. S6d), however, the water molecules hindered the main "Ca-O" interactions between the ASP and Ca atoms in HA. The DFT results were in accordance with our MD results and with previous reports in the literature[3]. Wang et al.[3] reported that water molecules on HA (110) surfaces could prevent BSA protein from moving close to the surface. Furthermore, there were water-bridged H-bonds between the amino acid residues, H_2O, and HA substrate.

In the water layer model, six H_2O molecules were initially placed on the HA (110) surface to form a hydrated layer, termed the WL model (Supplementary Fig. S6e). Then, the anionic ASP molecule was on the top of water molecules, whose configuration was also the same as that under vacuum. The interaction energy in the WL model was also less than that in the vacuum model (Supplementary Table S3). One water-bridged H-bond between the anionic ASP molecule, H_2O molecule and PO_4 in HA and one H-bond between the $-NH_2$ and H_2O were formed. In addition, electrostatic interactions occurred between the O atoms of ASP and the Ca atoms of

the HA surface (Supplementary Fig. S6f). The interaction distances of Ca and O were greater in the WL model than those in the vacuum, and the amount of charge transfer was also smaller in the WL model than those in the vacuum (Supplementary Table S3). Therefore, the adsorption energy became smaller in the WL model compared with the vacuum model.

In summary, our DFT study further confirms that water environments seriously affected the adsorption of amino acids on HA (110) surfaces, which is consistent with the results in our MD simulation and with the previous literature[4]. Guo et al.[4] found that H_2O molecules interact with the RGD or cap the adsorption sites of GO, thus, the adsorptions of RGD on the G and GO are weaker in the water system than those in vacuum.

Supplementary Table S3 Change in atomic charge and interaction energies of BMP-7 and flat HA surfaces in the vacuum, WE and WL models.

**Flat-HA-BMP-7 in the vacuum**				
	atoms	Before adsorption	After adsorption	△e
ASP27	Ca82	1.524	1.538	0.014
	Ca83	1.558	1.561	0.003
	O141	-0.362	-0.614	-0.252
	O142	-0.34	-0.757	-0.417
**Flat-HA-BMP-7 in the WL model**				
	atoms	Before adsorption	After adsorption	△e
ASP27	Ca82	1.509	1.519	0.010
	O141	-0.375	-0.388	-0.013
	O142	-0.463	-0.605	-0.142
	Total (Ha）	HA(110) (Ha）	ASP (Ha）	△E (eV)
HA(110)-ASP in the vacuum	-32851.67377	-32340.20965	-511.31393	-4.09
HA(110)-ASP in the WE model	-32851.57505	-32340.23478	-511.31232	-0.76
HA(110)-ASP in the WL model	-32851.67322	-32340.22717	-511.30859	-3.74

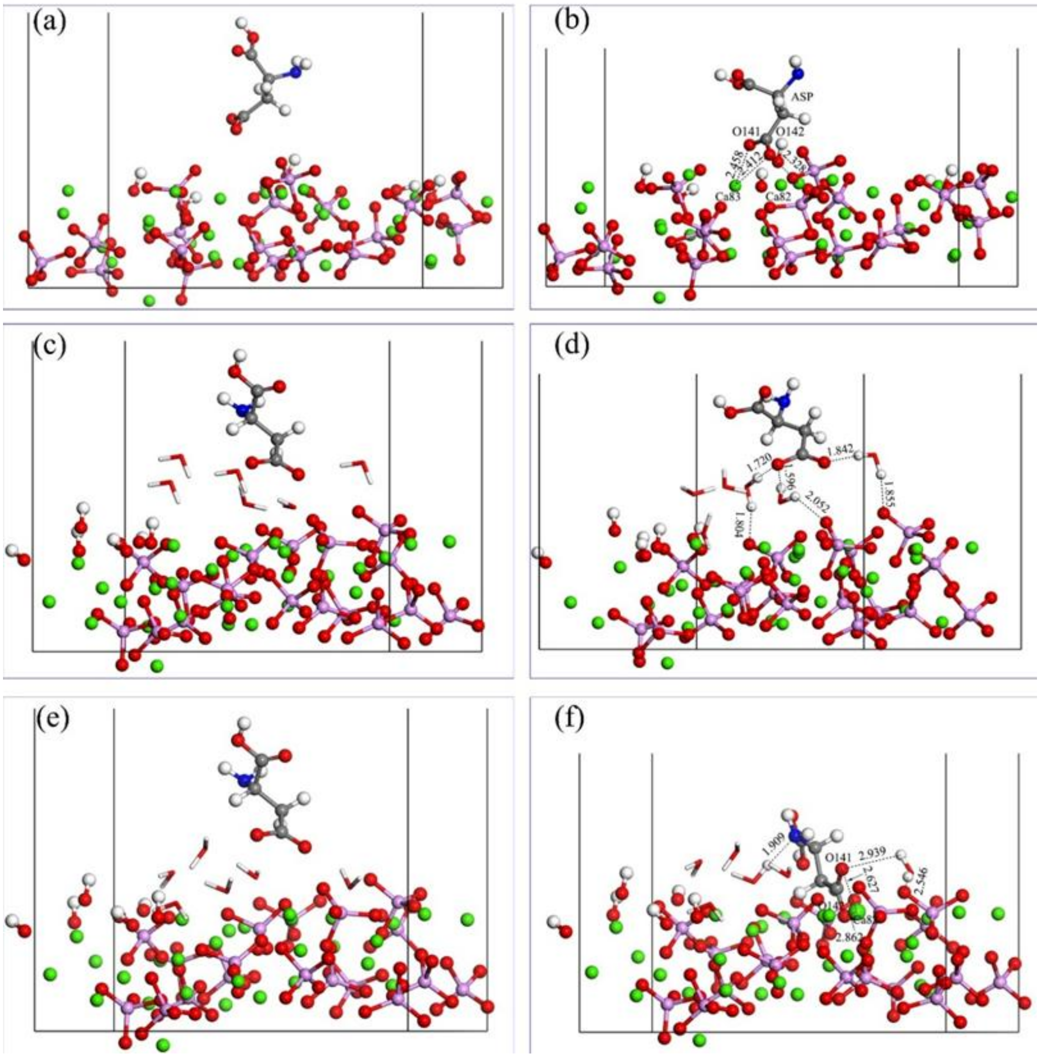

Supplementary Figure S6. Interaction of ASP with the Flat HA surface (**a**) and (**b**) in the vacuum; (**c**) and (**d**) in the WE model; (**e**) and (**f**) in this WL model, before and after geometry optimization, respectively.

Reference

1. Chiatti, F., Piane, M, D., Ugliengo, P. & Corno, M. Water at hydroxyapatite surfaces: the effect of coverage and surface termination as investigated by all-electron B3LYP-D* simulations. *Theor. Chem. Acc*. **135**, 1-15 (2016).
2. Astala, R. & Stott, M, J. First-principles study of hydroxyapatite surfaces and water adsorption. *Phys. Rev. B* **7**, 75427 (2008).

3. Wang, K. F., Wang, M. H., Wang, Q. G., Lu, X. & Zhang, X. D. Computer simulation of proteins adsorption on hydroxyapatite surfaces with calcium phosphate ions. *J. Eur. Ceram. Soc.* **37**, 2509-2520 (2017).
4. Guo, Y. N., Lu, X., Weng, J. & Leng, Y. Density functional theory study of the interaction of arginine-glycine-aspartic acid with graphene, defective graphene, and graphene oxide, *J. Phys. Chem. C* **117**, 5708-5717 (2013).
